# Iron-Based Contrast for the Heart: Clinical Promise of Ferumoxytol-Enhanced CMR

**DOI:** 10.1007/s12410-026-09618-5

**Published:** 2026-10-27

**Authors:** Arutyun Pogosyan, Yuehong Liu, Jasmine A. Patel, Mostafa Mahmoudi, Hang Xu, J. Paul Finn, Kim-Lien Nguyen

**Affiliations:** 1 Division of Cardiology, David Geffen School of Medicine at UCLA and VA Greater Los Angeles Healthcare System, Los Angeles, CA USA; 2 Department of Radiological Sciences, David Geffen School of Medicine at UCLA, Los Angeles, CA USA; 3 Department of Bioengineering, University of California, Los Angeles, CA USA; 4 David Geffen School of Medicine at UCLA, VA Greater Los Angeles Healthcare System, 11301 Wilshire Blvd, MC 111E, Los Angeles, CA 90073 USA; 5 Department of Radiological Sciences, University of California at Los Angeles, Peter V. Ueberroth Building Suite 3371, 10945 Le Conte Ave., Los Angeles, CA 90095-7206 USA

**Keywords:** Ferumoxytol, Cardiovascular magnetic resonance, Magnetic resonance imaging, USPIO, Contrast agents

## Abstract

**Purpose of Review:**

Ferumoxytol is an ultrasmall superparamagnetic iron oxide (USPIO) nanoparticle that was originally approved by the U.S. FDA for treating iron deficiency anemia in patients with chronic kidney disease. It has since emerged as an off-label, versatile intravascular contrast agent for cardiovascular magnetic resonance (CMR) imaging, and in 2025, received FDA approval for brain imaging. This review provides an overview of ferumoxytol-enhanced CMR applications primarily focused on the heart, outlines its key imaging and pharmacokinetic properties, and summarizes its diagnostic safety profile.

**Recent Findings:**

Unlike gadolinium-based contrast agents (GBCAs), which rapidly wash out from the circulation, ferumoxytol has high r_1_ and r_2_ relaxivities and remains confined to the intravascular compartment for prolonged periods. Once metabolized by the reticuloendothelial system, its iron core is incorporated into the body’s hematopoietic pathways. These unique properties enable steady-state and delayed-phase imaging, simplify complex imaging workflows, and facilitate accelerated multidimensional cardiac imaging methods. Emerging applications for ferumoxytol-enhanced CMR include myocardial perfusion assessment, multidimensional imaging, vascular and hemodynamic evaluation, and comprehensive structural heart disease imaging.

**Summary:**

Ferumoxytol expands the clinical applications of CMR by enabling imaging techniques that are not feasible with conventional GBCAs and broadening imaging options for patients in whom GBCA administration may be less desirable based on individualized clinical risk-benefit assessment, including selected patients with renal insufficiency, very young children, and pregnant women.

**Graphical Abstract:**

**Figure:**
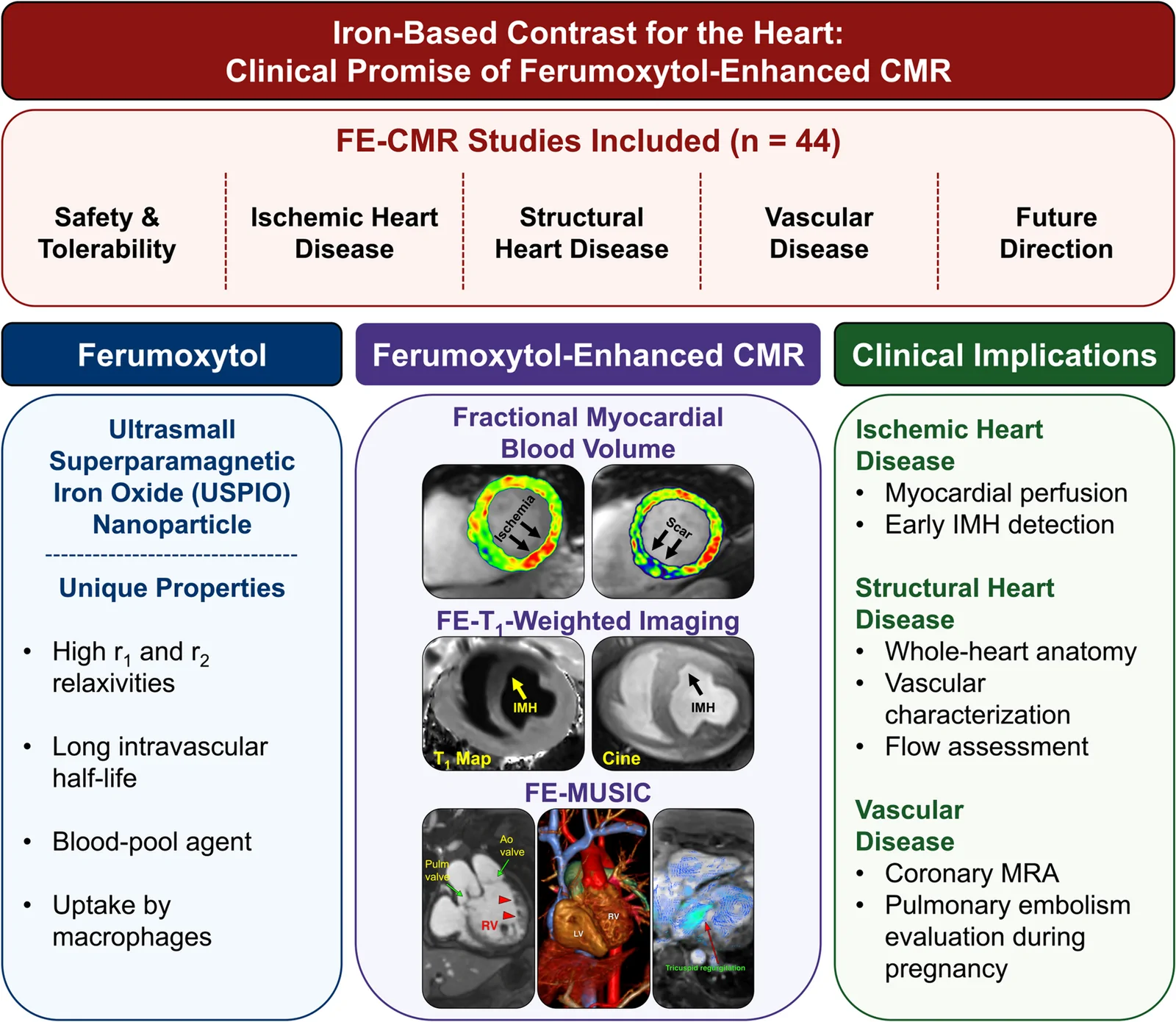

## Introduction

Cardiovascular magnetic resonance (CMR) is an established imaging modality for the diagnosis and management of a broad range of cardiovascular diseases, offering detailed insights into anatomy, tissue composition, cardiac function, and blood flow. Contrast agents play a crucial role in expanding the diagnostic capabilities of CMR. These agents contain paramagnetic ions with unpaired electrons that alter the longitudinal (T_1_) and/or transverse (T_2_/T_2_*) relaxation properties of surrounding hydrogen protons, thereby enhancing tissue contrast and enabling more accurate tissue characterization.

Gadolinium-based contrast agents (GBCAs) remain the most widely used MRI contrast agents, with an estimated 50 million doses administered annually worldwide [1]. Over the past four decades, research has established their role and favorable safety profile in the diagnosis and management of cardiovascular disease. In 2006, reports linking linear GBCAs to nephrogenic systemic fibrosis (NSF), a rare but potentially fatal condition associated with the release of toxic free Gd^3+^ ions, raised safety concerns in patients with renal impairment [2, 3], leading to reduction in GBCA dose and adoption of more stable macrocyclic formulations with lower risk of free Gd^3+^ dissociation [1, 4]. However, gadolinium is a rare earth metal with no known physiologic role in normal human metabolism. More recent reports of its deposition within the brain [5–8] and introduction into the biosphere [9] have prompted renewed safety and environmental concerns. In addition, due to their relatively small molecular weight (500–1000 Da), GBCAs rapidly extravasate into the interstitial space following intravascular administration, resulting in a limited blood-pool imaging window. This imposes trade-offs among spatial resolution, temporal resolution, and anatomic coverage in CMR, which may compromise image quality and complicate perfusion modeling. In this context, increasing attention has shifted toward alternative contrast agents capable of overcoming some of the inherent limitations of conventional GBCAs.

Ferumoxytol has emerged as a promising MRI contrast agent with several unique properties that distinguish it from conventional GBCAs. Ferumoxytol is a specific and potent blood-pool contrast agent that carries no risk of contrast-induced nephropathy, making it particularly attractive for elderly patients, pregnant women, and those with renal impairment. Since its approval by the U.S. Food and Drug Administration (FDA) in 2009 for the treatment of iron deficiency anemia in adults with chronic kidney disease (CKD), off-label diagnostic use of ferumoxytol (Feraheme; Azurity Pharmaceuticals, Waltham, Massachusetts) has gained increasing traction among researchers and clinicians, particularly in CMR. As of October 2025, ferumoxytol under the trade name Ferabright (Azurity Pharmaceuticals, Woburn, Massachusetts) has received FDA approval for brain tumor MRI.

**Fig. 1:**
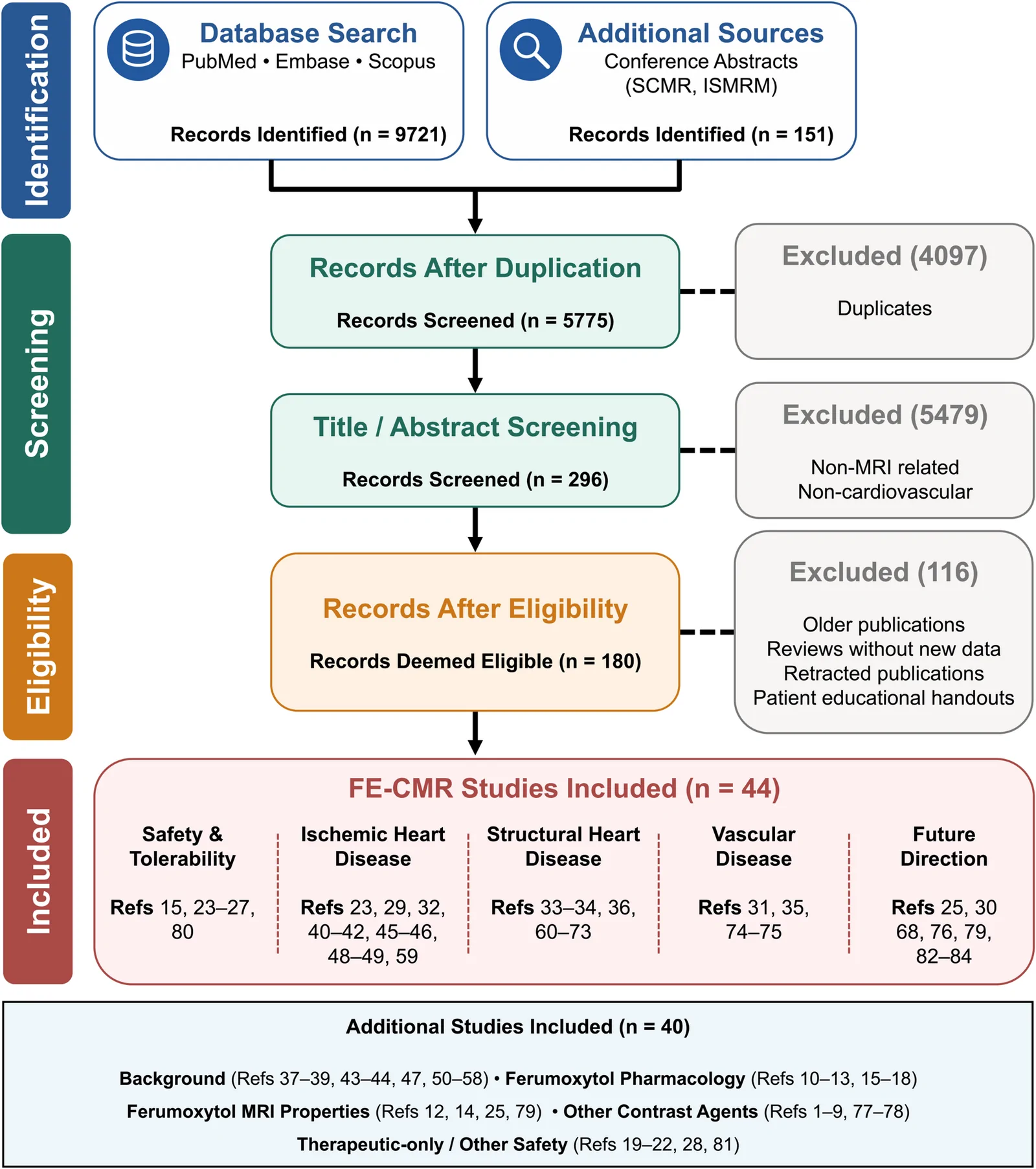
Study selection flow diagram. Flow diagram summarizing the literature search, screening, eligibility assessment, and inclusion process for studies evaluating ferumoxytol-enhanced cardiovascular magnetic resonance imaging (FE-CMR). Records were identified through PubMed, Embase, Scopus, and conference abstracts (SCMR and ISMRM) using the search terms: “ferumoxytol,” "ferumoxytol-enhanced MRI,” and “ferumoxytol-enhanced CMR.” Included studies were categorized into safety and tolerability, ischemic heart disease, vascular disease, structural heart disease, and future directions. Additional background references were also included. *SCMR*, Society of Cardiovascular Magnetic Resonance; *ISMRM*, International Society of Magnetic Resonance in Medicine

In this review, we summarize the key imaging attributes and diagnostic safety profile of ferumoxytol and review its applications in ferumoxytol-enhanced CMR (FE-CMR), with a primary focus on the heart. This narrative review was informed by a systematic literature search of published articles in PubMed, EMBASE, and Scopus, along with scientific abstracts from the Society of Cardiovascular Magnetic Resonance and the International Society for Magnetic Resonance in Medicine. Search terms included “ferumoxytol,” “ferumoxytol-enhanced MRI,” and “ferumoxytol-enhanced CMR” (Fig. 1). Identified records were screened by title and abstract for relevance, followed by full-text assessment of potentially eligible publications. Studies addressing the diagnostic use, safety, or cardiovascular imaging applications of ferumoxytol were considered for inclusion. Priority was given to original research, recent expert reviews, and publications within the last five years (2021–2026). Non-cardiovascular studies, publications before 2015, and reviews without new data were excluded from the core FE-CMR study selection. The literature search was last updated in May 2026. A total of 44 studies published between 2015 and 2026 were included, of which 33 were published during 2021–2026. Additional references were included as needed to provide background on ferumoxytol pharmacology, MRI properties, other contrast agents, and therapeutic safety.

## Pharmacodynamics

Table 1 summarizes iron-based MRI contrast agents. Ferumoxytol is currently the only clinically available iron-based MRI contrast agent. As an ultrasmall superparamagnetic iron oxide (USPIO) nanoparticle, ferumoxytol is composed of an iron oxide core and an outer carbohydrate shell. Its magnetic properties are determined by the superparamagnetic iron oxide core, while the surrounding carboxymethyl-dextran coating influences its safety, pharmacokinetics, intravascular stability, circulation time, and biodistribution. The coating maintains a near-neutral surface charge that limits the release of potentially harmful labile iron and enhances its safety profile [10].

**Table 1:** Overview of intravenous iron-based contrast agents for magnetic resonance imaging

Generic Name	Trade Name	Hydrodynamic Diameter (nm)	MRI Properties	Primary Application
Ferucarbotran^2, ‡^	Resovist (Japan)	45–60	Predominantly T_2_ / T_2_* shortening with some T_1_ effect at low concentration	Liver imaging
Feruglose^3^	Clariscan	11–20	Predominantly T_1_ shortening at lower concentrations	Vascular imaging
Ferumoxide^1, 2, †, ‡^	Feridex IV (USA); Endorem (EU)	80–150	Strong T_2_ / T_2_* effects	Liver and spleen imaging
Ferumoxtran^3^	Combidex (USA); Sinerem (EU)	20–50	Early T_1_ shortening with delayed T_2_ / T_2_* effects	Lymph node and inflammatory imaging
Ferumoxytol^1, 2, ‡^	Feraheme (USA); Ferabright (USA)	17–30	Strong T_1_ and T_2_ / T_2_* effects	Vascular, cardiovascular, neurovascular, and oncologic imaging

^1^ US Food and Drug Administration (FDA) approval

^2^ European Medicines Agency

^3^ Investigational

^†^ Withdrawn from the US market

^‡^ Withdrawn from EU market

**Fig. 2:**
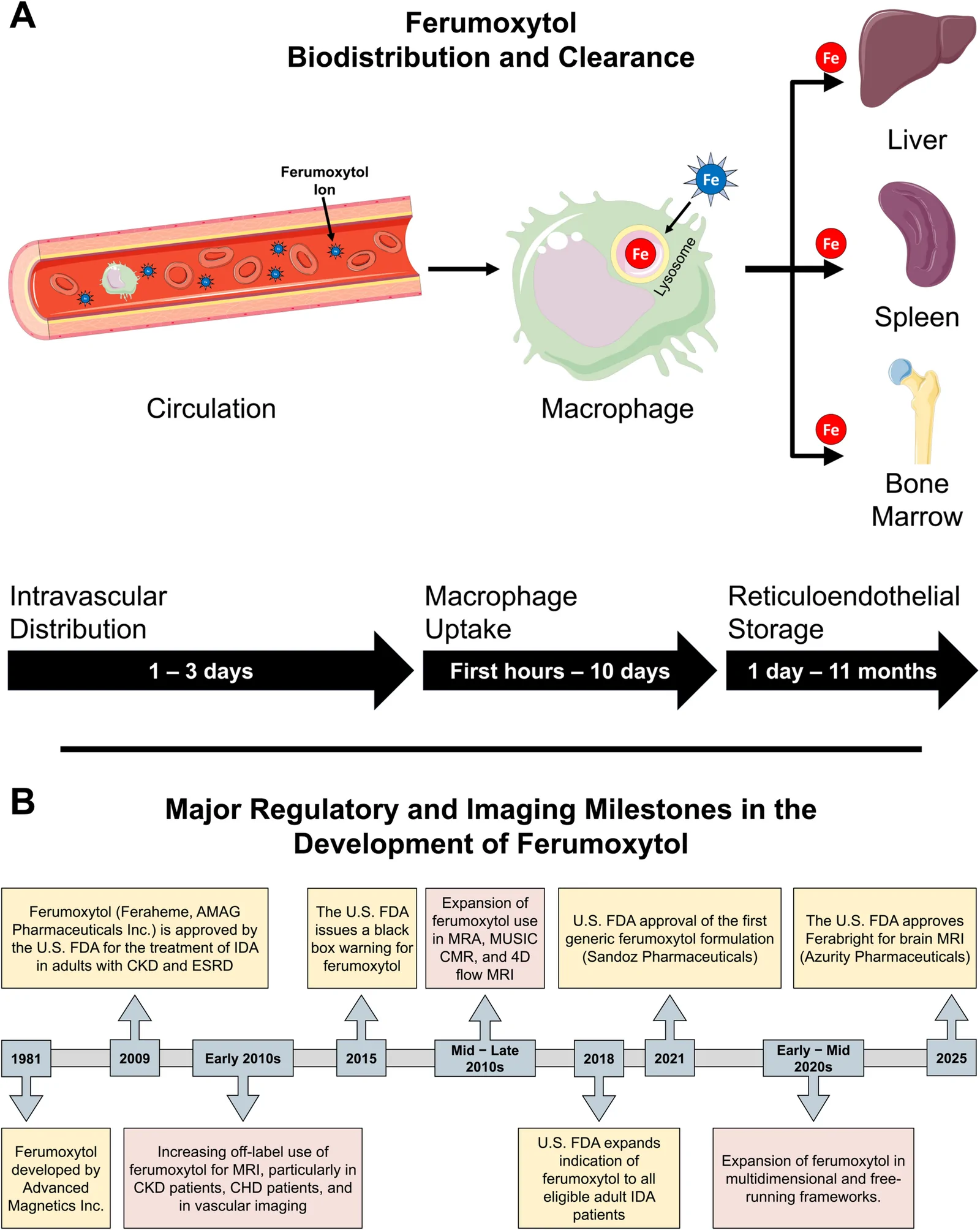
Pharmacologic and Imaging Overview of Ferumoxytol. (**A**) Schematic illustration of ferumoxytol biodistribution and clearance. Following intravenous administration, ferumoxytol remains within the intravascular space for a prolonged period before gradual uptake by macrophages of the reticuloendothelial system, with subsequent storage and metabolism in the liver, spleen, and bone marrow. Adapted from Servier Medical Art (https://smart.servier.com), licensed under CC BY 4.0 (https://creativecommons.org/licenses/by/4.0/). (**B**) Timeline highlighting major regulatory and imaging milestones in the development of ferumoxytol, including initial FDA approval for iron deficiency anemia, subsequent safety-related regulatory actions, expansion of off-label diagnostic imaging applications, and recent approval of an MRI-specific ferumoxytol formulation. *CHD*, congenital heart disease; *CKD*, chronic kidney disease; *CMR*, cardiovascular magnetic resonance; *ESRD*, end-stage renal disease; *Fe*, ferumoxytol; *IDA*, iron deficiency anemia; *MRA*, magnetic resonance angiography; *MRI*, magnetic resonance imaging; *MUSIC*, multiphase steady-state imaging with contrast; *U.S. FDA*, U.S. Food and Drug Administration

A key consideration for any contrast agent is its route of elimination. Whereas GBCAs are cleared by the kidneys through glomerular filtration, ferumoxytol clearance from the blood is independent of renal function and occurs through uptake by hepatic and splenic macrophages within the mononuclear phagocyte system (MPS) over a period of several days (Fig. 2A) [11, 12]. Following macrophage internalization of USPIO particles, iron is released from the particle core within intracellular lysosomes and subsequently incorporated into the body’s physiologic pathways. Unlike gadolinium, iron is an essential element. The liberated iron is ultimately stored in the liver, spleen, and bone marrow, where it is recycled for hemoglobin synthesis over the ensuing weeks to months [11, 12]. Because iron within the reticuloendothelial system can alter liver T_2_ signal characteristics on MRI examinations for weeks [11], a history of intravenous iron therapy should be considered when interpreting relevant MRI studies.

## Blood Pool Properties and Imaging Advantages

Major regulatory and developmental milestones for ferumoxytol are summarized in Fig. 2B. As a complementary contrast agent to GBCAs, ferumoxytol remains largely intravascular with minimal extravasation, resulting in a low volume of distribution and a stable blood-pool concentration until MPS uptake. Its large molecular size (~ 750 kDa) limits transendothelial diffusion and contributes to an intravascular half-life exceeding 14 h [13]. Ferumoxytol can support both dynamic (first-pass) and steady-state imaging, while enabling repeated image acquisition if the initial study is degraded by patient movement or respiratory motion artifacts. In contrast, conventional GBCAs rapidly distribute into the extracellular space after the arterial first pass, resulting in a short vascular imaging window.

Like most conventional exogenous contrast agents, ferumoxytol alters image contrast by shortening the relaxation times of surrounding water protons, thereby increasing signal differences relative to native tissue. Compared to GBCAs, ferumoxytol exhibits approximately 3–5-fold higher r_1_ relaxivity and 7–15-fold higher r_2_ relaxivity [14]. Owing to its combined T_1_ shortening and strong T_2_/T_2_* susceptibility effects, ferumoxytol can generate either positive (T_1_-weighted) or negative (T_2_/T_2_*-weighted) contrast depending on imaging parameters, supporting structural imaging and sensitivity to susceptibility-driven pathophysiologic changes [10, 14].

## Dosing, Administration, and Safety Considerations

Typical ferumoxytol doses for diagnostic use range from 2.0 to 7.5 mg/kg, with most cases using 3.0 to 4.0 mg/kg in both pediatric and adult populations [15–17]. This is substantially lower than the standard cumulative therapeutic dose of 1020 mg administered for the treatment of anemia, equivalent to 14.6 mg/kg in a 70-kg adult. In accordance with FDA recommendations, ferumoxytol is typically diluted with normal saline to a final concentration not exceeding 10 mg/ml and administered as a slow intravenous infusion, generally at rates up to 0.5 mg/s or over approximately 8 min [16–18].

Table 2 summarizes recent studies and their reported safety findings for the diagnostic use of ferumoxytol. Serious adverse events are exceedingly rare and have primarily been reported in the therapeutic setting [19, 20]. The Fishbane reaction is the most common adverse event, representing a self-limited, non-immune-mediated syndrome characterized by myalgias, arthralgias, headache, and back pain, without features of anaphylaxis such as wheezing, stridor, periorbital edema, or persistent hypotension [21, 22]. Recognition is important to distinguish it from true anaphylaxis and avoid inappropriate intervention or termination of the study.

**Table 2:** Selected safety studies of ferumoxytol in diagnostic imaging

Year	Population	Ferumoxytol dose	Application	Hemodynamic monitoring	Adverse events	Reference
2026	*N* = 72 (20 – 88 years)	3–4 mg/kg	Regadenoson stress FE-CMR in healthy and IHD	HR, MAP	Mild: 5 (6.9%) Moderate: 1 (1.4%) Severe: 0 (0%)	Pogosyan et al. [23]
2023	*N* = 30 (54 – 70 years)	3 mg/kg	FE Coronary MRA	HR, MAP, SpO_2_	Mild: 0 (0%) Moderate: 0 (0%) Severe: 0 (0%)	Dong et al. [24]
2022	*N* = 390 (6 days – 62 years)	2–3 mg/kg	FE-MRA and 4D-flow	HR, SBP, DBP, SpO_2_	Mild: 14 (3.6%) Moderate: 5 (1.3%) Severe: 0 (0%)	Dasi et al. [25]
2019	*N* = 3215 patients; *N* = 4240 injections (1 day – 96 years)	1–11 mg/kg	Various FE-MRI	HR, MAP, SpO_2_	Mild: 75 (1.8%) Moderate: 8 (0.2%) Severe: 0 (0%)	Nguyen et al. [26]
2016	*N* = 217 (3 days – 94 years)	4 mg/kg	Various FE-MRI	HR, SBP, DBP, SpO_2_, end-tidal CO_2_	Mild: 2 (1.8%) Moderate: 0 (0%) Severe: 0 (0%)	Nguyen et al. [15]

*CMR* cardiovascular magnetic resonance, *DBP* diastolic blood pressure, *FE* ferumoxytol-enhanced, *HR* heart rate, *IHD* ischemic heart disease, *MAP* mean arterial pressure, *MRI* magnetic resonance imaging, *MRA* magnetic resonance angiography, *SBP* systolic blood pressure, *SpO*_*2*_ peripheral oxygen saturation

Overall, accumulating evidence indicates that the diagnostic use of ferumoxytol is well tolerated. The retrospective, multicenter FeraSafe Registry study, one of the largest published diagnostic safety studies to date, included 3215 patients who underwent a total of 4240 ferumoxytol administrations for MRI between 2003 and 2018 (dose range: 1–11 mg/kg) [26]. No severe, life-threatening, or fatal adverse events were reported. Mild reactions were uncommon (1.8%), and moderate reactions were rare (0.2%). Moreover, among 887 patients with available hemodynamic data, no systematic changes in mean arterial pressure or heart rate were identified. Even with the concurrent use of a vasodilator during stress testing, a recent prospective study demonstrated only minor, transient reductions in mean arterial pressure during ferumoxytol-enhanced cardiac stress imaging with regadenoson (pre-ferumoxytol at rest: 86.0 ± 11.4 mm Hg vs. post-ferumoxytol at rest: 89.7 ± 13.8 mm Hg vs. post-ferumoxytol at peak hyperemia: 86.4 ± 13.3 mm Hg) [23]. Although severe adverse reactions at diagnostic doses are exceedingly rare, isolated cases of anaphylaxis have been reported, including in a medically complex pediatric patient with a YARS genetic mutation and prior deceased-donor liver transplantation who developed severe anaphylaxis requiring emergent intubation after receiving 4 mg/kg ferumoxytol for MRI evaluation of the renal vasculature, underscoring that serious hypersensitivity reactions remain possible even at imaging doses [27]. As with all intravenous iron formulations, ferumoxytol should be administered by personnel trained in the recognition and management of infusion-related reactions. In a recent expert consensus guideline published in 2024, pre-infusion assessment is recommended to evaluate the risk of infusion reactions, along with appropriate monitoring before and after administration [28].

## Current and Emerging Applications of FE-CMR

Table 3 summarizes key FE-CMR publications from 2021 to 2026, highlighting recent advances in cardiovascular applications. The clinical maturity of FE-CMR varies across applications. Ferumoxytol-enhanced angiographic and structural imaging has been increasingly adopted for clinically indicated examinations, particularly in patients with congenital heart disease (CHD) or renal impairment; whereas, applications involving myocardial perfusion, intramyocardial hemorrhage and inflammation, and multidimensional physiologic imaging remain predominantly investigational and are supported by early clinical or preclinical studies. The following sections highlight both established clinical applications and emerging areas of FE-CMR development.

**Table 3:** Selected key publications in ferumoxytol-enhanced cardiovascular magnetic resonance (2021–2026)

Year	Technique	Key findings	Reference
2026	Regadenoson stress FE-CMR	Regadenoson stress FE-CMR was generally well tolerated, produced only modest hemodynamic changes, and revealed progressively blunted FE-T_1_ reactivity in remote, ischemic, and infarcted myocardium relative to healthy tissue.	Pogosyan et al. [23]
2026	Hyperacute intramyocardial hemorrhage imaging	Demonstrated the feasibility of T_1_-weighted FE-CMR for hyperacute detection of intramyocardial hemorrhage following ischemia-reperfusion injury in a swine model.	Pogosyan et al. [29]
2025	Coronary microvascular network synthesis	Developed a multiscale framework integrating FE coronary MRI and fMBV mapping to generate patient-specific coronary microvascular networks.	Mahmoudi et al. [30]
2025	Steady-state thoracic FE-MRA	Single breath-held steady-state FE-MRA provided equivalent or superior thoracic vascular visualization compared with conventional dynamic gadolinium-enhanced MRA, without bolus timing requirements and with a simplified workflow.	Bedayat et al. [31]
2025	Cine DENSE strain imaging	Cine DENSE demonstrated high reproducibility of global and segmental circumferential strain measurements before and after ferumoxytol administration at 3T, supporting the feasibility of myocardial strain imaging during FE-CMR.	Li et al. [32]
2025	Accelerated free-running whole-heart angiography	XD-MC-SIMBA enabled highly accelerated FE free-running whole-heart angiography in CHD patients, supporting diagnostic-quality imaging in acquisition times as short as 3 min or less.	Romanin et al. [33]
2024	ROCK-MUSIC with low-rank reconstruction	Integration of low-rank tensor reconstruction with FE ROCK-MUSIC improved cardiac and vascular image quality while enabling cardiorespiratory phase-resolved imaging in pediatric CHD patients.	Zhao et al. [34]
2023	FE coronary MRA	FE coronary MRA demonstrated good diagnostic performance and excellent safety in the diagnosis of significant coronary artery stenosis.	Dong et al. [24]
2022	Pulmonary FE-MRA during pregnancy	FE pulmonary MRA provided a radiation- and gadolinium-free alternative for pulmonary embolism evaluation during pregnancy, with good image quality and high diagnostic confidence.	Starekova et al. [35]
2021	4D MUSIC whole-heart imaging	4D FE MUSIC CMR in pediatric CHD demonstrated high image quality and diagnostic confidence while significantly reducing acquisition time compared with conventional MRI protocols.	Nguyen et al. [36]

*CHD* congenital heart disease, *CMR* cardiovascular magnetic resonance, *DENSE* displacement encoding with stimulated echoes, *FE* ferumoxytol-enhanced, *fMBV* fractional myocardial blood volume, *MRI* magnetic resonance imaging, *MRA* magnetic resonance angiography, *ROCK-MUSIC* rotating Cartesian k-space multiphase steady-state imaging with contrast enhancement, *XD-MC-SIMBA* extra-dimensional motion-compensation similarity-based angiography

### Myocardial Perfusion Imaging

Patients with CKD exhibit a higher prevalence of ischemic heart disease (IHD) and are more likely to present with silent ischemia [37], which may confer increased risk of major cardiovascular events [38]. While invasive coronary angiography remains the reference standard for diagnosing obstructive coronary artery disease, CMR techniques such as first-pass stress perfusion are increasingly being utilized as a noninvasive alternative, providing simultaneous assessment of myocardial perfusion, function, and viability, without ionizing radiation. FE-CMR holds the potential to extend this capability by enabling noninvasive perfusion assessment in patients where GBCA use may be less desirable or pharmacologic stress is not feasible.

**Fig. 3:**
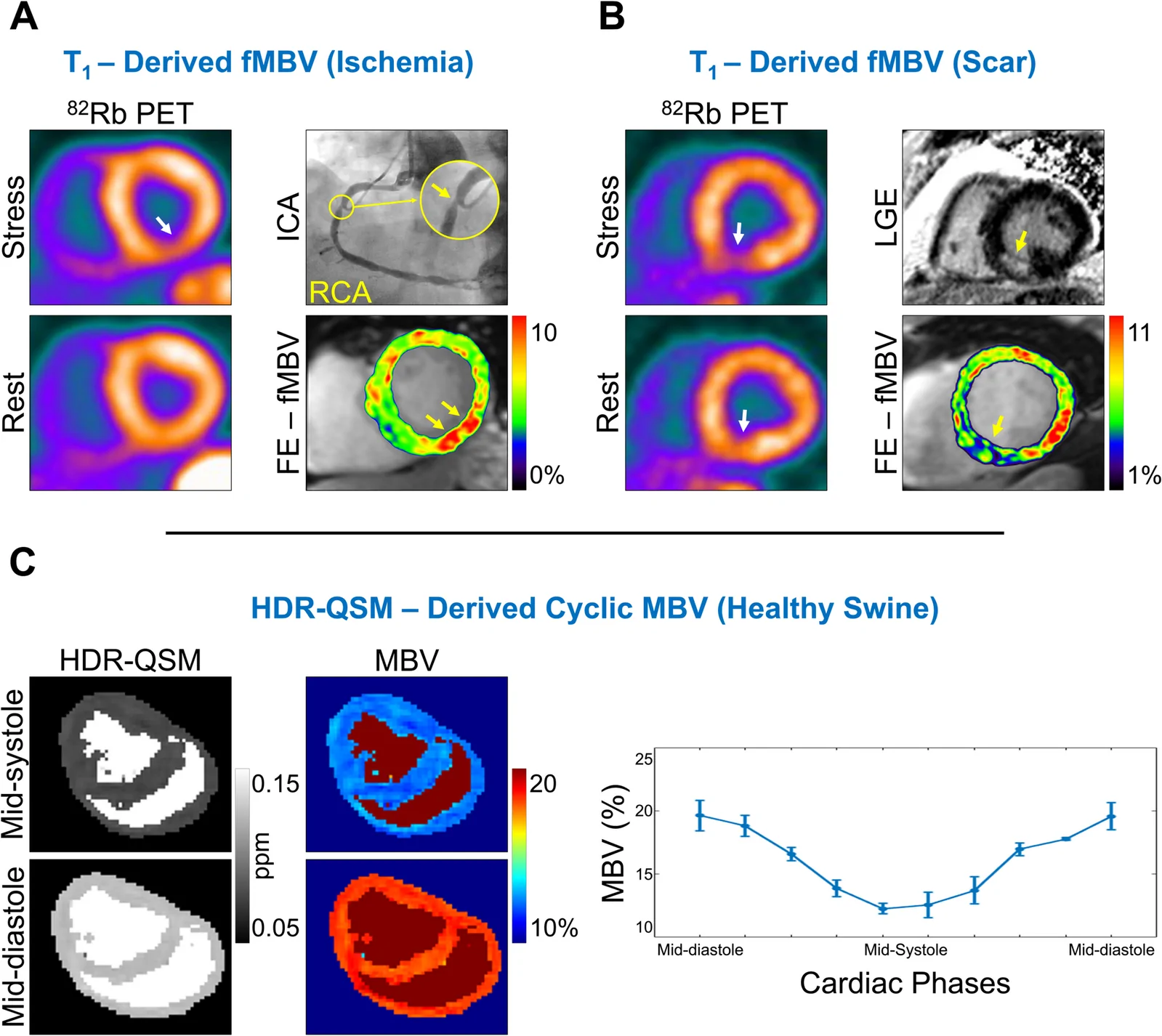
Ferumoxytol-enhanced myocardial blood volume imaging techniques. (**A**) Rest and stress short-axis rubidium-82 (^82^Rb) cardiac positron emission tomography (PET) images from a representative patient with myocardial ischemia demonstrates a reversible perfusion defect in the proximal inferior wall during stress (white arrow). Corresponding invasive coronary angiography (ICA) demonstrates right coronary artery stenosis (yellow arrow). The corresponding short-axis ferumoxytol-enhanced T_1_-derived fractional myocardial blood volume (FE-fMBV) map demonstrates elevated fMBV within the affected myocardial territory (yellow arrows). (**B**) Rest and stress short-axis ^82^Rb cardiac PET images from a representative case of myocardial infraction demonstrating a fixed perfusion defect within the mid-inferior and inferoseptal walls (white arrows). Corresponding late gadolinium enhancement (LGE) confirms myocardial infarction, and matching short-axis T_1_-derived FE-fMBV map demonstrates reduced fMBV within the infarcted myocardium (yellow arrow). (**C**) Cardiac motion–resolved high-dynamic-range quantitative susceptibility mapping (HDR-QSM) images acquired at mid-systole and mid-diastole in a healthy swine, corresponding myocardial blood volume (MBV) maps, and quantified cyclic MBV measurements demonstrate the feasibility of QSM-derived MBV imaging and the expected physiological variation in MBV across the cardiac cycle, with higher MBV during diastole than systole. Panel C adapted with permission from [42]

Fractional myocardial blood volume (fMBV) reflects the fraction of myocardial tissue occupied by intramyocardial blood. In the setting of coronary stenosis, autoregulatory responses within the epicardial and microvascular circulation increase myocardial blood volume (MBV) [39]. As a purely intravascular agent with high r_1_ relaxivity, ferumoxytol enhances the sensitivity of T_1_ to these intravascular changes, enabling voxel-wise estimation of fMBV from myocardial and blood T_1_ at contrast steady state. When combined with a two-compartment water-exchange model, ferumoxytol-enhanced T_1_ mapping following incremental intravenous doses allows pixel-wise estimation of fMBV by exploiting the relationship between the intravascular contrast agent concentration and the change in myocardial longitudinal relaxation rate [40]. fMBV has been shown to be effective at differentiating ischemic from remote myocardium in preclinical studies [41], and may serve as a noninvasive biomarker for patients with renal impairment or contraindications to vasodilator stress (Fig. 3A and B).

Throughout the cardiac cycle, fMBV fluctuates due to cyclic changes in intramyocardial pressure, myocyte contraction, and vascular tethering [43]. Characterizing these cyclic variations may provide additional insight into ischemia-related alterations in myocardial perfusion. MR Multitasking provides a framework to conceptualize physiological motions and other dynamic processes as multiple time dimensions that can be resolved simultaneously via low-rank tensor decomposition, allowing motion-resolved quantitative parametric mapping without ECG triggering or breath-holds [44]. When combined with ferumoxytol’s steady-state blood-pool enhancement, Multitasking enables dynamic, cardiac-phase-resolved CMR. Huang et al. recently demonstrated significant cyclic differences in MBV between systolic and diastolic phases in healthy myocardium using motion-resolved, high-dynamic-range quantitative susceptibility mapping (HDR-QSM) with ferumoxytol in a swine model (Fig. 3C), while infarcted myocardium exhibited significantly reduced MBV following induction of acute myocardial infarction [42]. Unal et al. developed a hybrid 2D/3D ferumoxytol-enhanced myocardial T_1_-tracking approach capable of capturing cyclic intramyocardial blood volume dynamics [45]. More recently, the concept of dynamic MBV was extended using ferumoxytol-enhanced T_1_-based Multitasking to generate cardiac phase–resolved fMBV maps in both healthy subjects and patients with IHD [46]. In healthy volunteers, fMBV increased from end-systole to end-diastole, consistent with established physiology [47]. In contrast, infarcted segments in patients with IHD exhibited reduced fMBV with blunted cyclic variation relative to healthy myocardium.

**Fig. 4:**
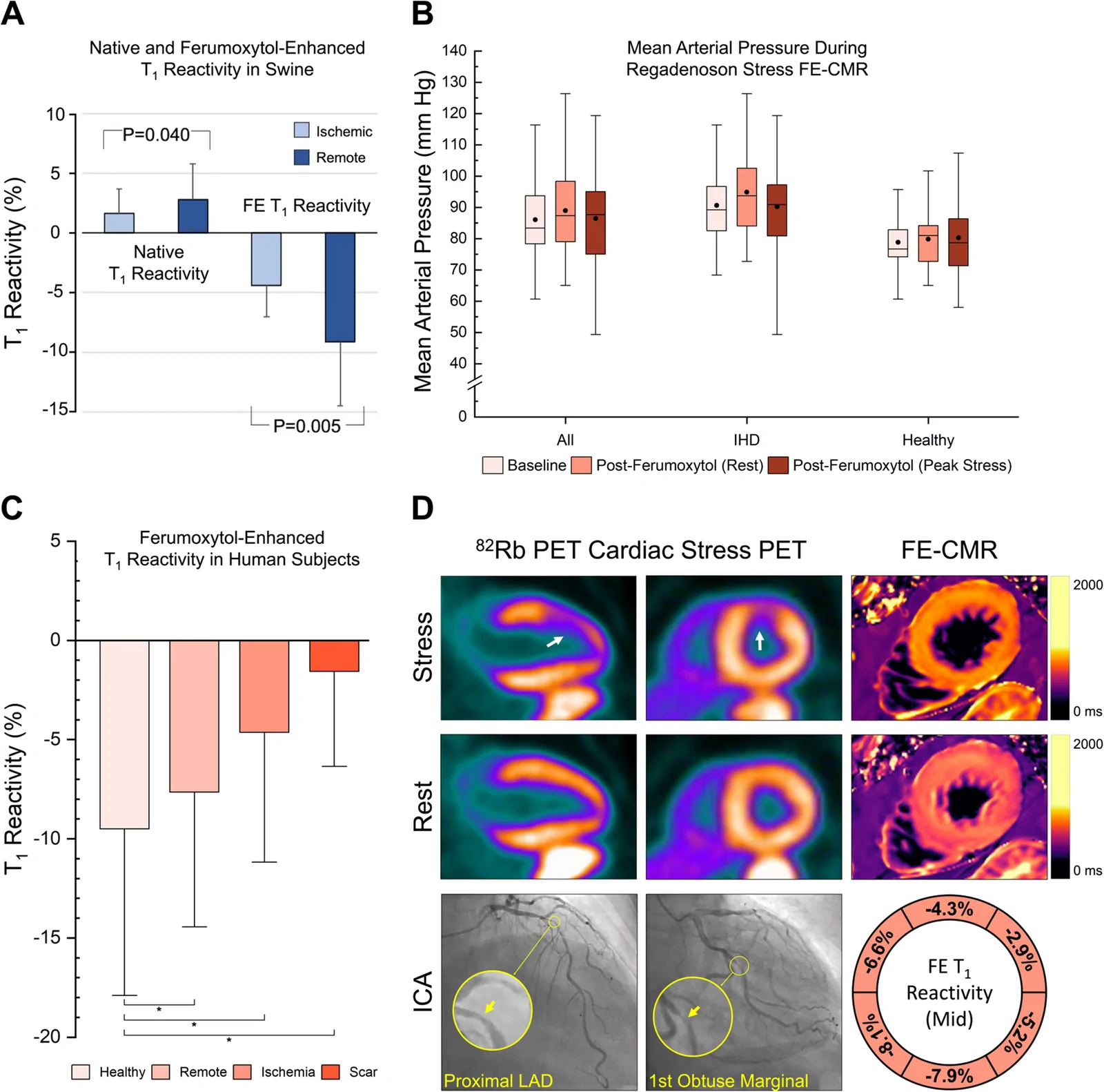
Native and ferumoxytol-enhanced T_1_ reactivity in ischemic heart disease. (**A**) Native and ferumoxytol-enhanced (FE) T_1_ reactivity in a swine model of ischemia–reperfusion injury. Compared with native T_1_ reactivity, FE-T_1_ reactivity demonstrated a larger dynamic range and greater separation between remote and ischemic myocardium. Adapted with permission from [49]. (**B**) Mean arterial pressure (MAP) changes during regadenoson stress ferumoxytol-enhanced cardiovascular magnetic resonance (FE-CMR). Box plots demonstrate MAP at baseline, post-ferumoxytol at rest, and post ferumoxytol at peak stress in 44 patients with ischemic heart disease (IHD) and 28 healthy volunteers. Boxes represent the interquartile range (25th–75th percentile), center lines indicate the median, dots indicate the mean, and whiskers represent minimum and maximum values. (**C**) Bar graph depicting FE-T_1_ reactivity in healthy volunteers and patients with IHD, showing progressively blunted responses across remote, ischemic, and scarred myocardium relative to healthy tissue. Error bars indicate standard deviation. Asterisks (*) denote statistically significant differences (p-value < 0.05). (**D**) Representative FE-CMR stress T_1_ reactivity maps in a patient with multivessel coronary artery disease demonstrating blunted T_1_ reactivity within the anterior and anterolateral walls, corresponding to a reversible perfusion defect in the same region on stress rubidium-82 (^82^Rb) cardiac positron emissions tomography (PET), and corroborated by coronary stenoses within the proximal left anterior descending (LAD) and first obtuse marginal arteries on invasive coronary angiography (ICA). Panels B–D adopted with permission from [23]

In addition to fMBV quantification, FE-CMR can be used to assess vasodilator-induced changes in myocardial T_1_ as a surrogate for vascular reactivity and myocardial vasodilatory reserve [23, 48, 49] (Fig. 4). Native T_1_ reactivity, which reflects the percent change in non-contrast myocardial T_1_ between rest and peak vasodilator-induced stress, has been proposed as a contrast-free method to differentiate between healthy, ischemic, and infarcted myocardium by detecting alterations in myocardial water content [50, 51]. However, its clinical utility is limited by a relatively narrow dynamic range and sensitivity to confounding factors [52]. In comparison, ferumoxytol-enhanced T_1_ mapping has been proposed to amplify sensitivity to intravascular volume changes, thereby extending the dynamic range of T_1_ response during vasodilator stress. In this framework, changes in myocardial T_1_ reflect alterations in intramyocardial blood volume in response to pharmacologic vasodilation, providing a functional measure of vascular reserve. Early preclinical studies have shown that ferumoxytol-enhanced T_1_ reactivity (∆FE-T_1_) during vasodilator stress can differentiate remote from ischemic myocardium, with ischemic regions exhibiting attenuated responses consistent with impaired vasodilatory reserve [49]. Compared with native T_1_ reactivity, ∆FE-T_1_ provided improved diagnostic performance for detecting ischemia (area under the curve, 0.81–0.92 vs. 0.72–0.79) [49]. These findings were subsequently extended to healthy human subjects and patients with known IHD, demonstrating progressively attenuated responses in remote, ischemic, and scarred regions relative to healthy myocardium, while maintaining an overall favorable safety profile [23] (Fig. 4).

### Imaging of Intramyocardial Hemorrhage and Inflammation

**Fig. 5:**
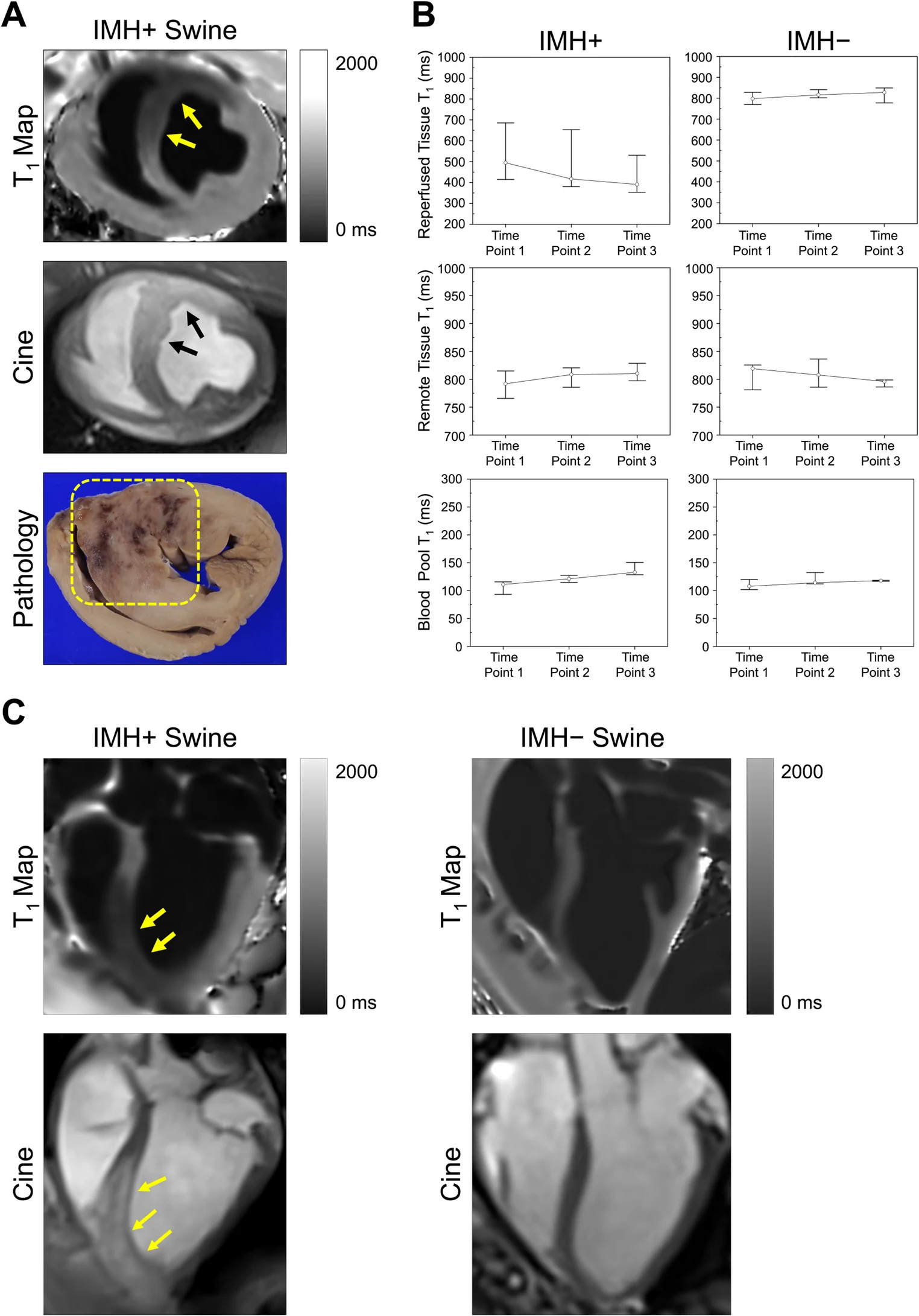
Ferumoxytol-enhanced cardiovascular magnetic resonance for detection of hyperacute intramyocardial hemorrhage following ischemia–reperfusion injury. (**A**) Representative short-axis T_1_ map, cine image, and gross pathology specimen from an intramyocardial hemorrhage-positive (IMH+) swine. Ferumoxytol-enhanced T_1_ mapping demonstrates focal regions of markedly reduced T_1_ within the reperfused myocardium (yellow arrows), corresponding to signal abnormalities on cine imaging (black arrows) and gross hemorrhagic involvement on pathology. (**B**) Serial T_1_ measurements across three time points within 6 h of reperfusion in IMH + and IMH-negative (IMH−) swine. IMH+ animals demonstrated persistently reduced T_1_ values within reperfused myocardium relative to IMH− animals, while remote myocardial and blood pool T_1_ values remained comparatively stable. (**C**) Representative long-axis ferumoxytol-enhanced T_1_ maps and cine images from IMH + and IMH− swine. Regions of hemorrhagic myocardium in IMH+ animals (yellow arrows) demonstrate marked T_1_ shortening compared with nonhemorrhagic reperfused myocardium in IMH− animals. Adapted with permission from [29]

A major complication of acute myocardial infarction is intramyocardial hemorrhage. Although timely reperfusion remains the standard of care, restoration of coronary blood flow can paradoxically induce ischemia–reperfusion injury, with downstream consequences such as intramyocardial hemorrhage, which has been recognized as a key indicator of poor long-term prognosis [53, 54]. T_2_* cardiac MRI is the reference standard for detecting intramyocardial hemorrhage after myocardial infarction by exploiting susceptibility effects from iron released during heme degradation [55]. However, because these metabolites accumulate over 1–3 days, its sensitivity within the first 24 h of reperfusion remains limited despite histopathologic evidence of earlier hemorrhage [56–58]. To address this gap, our group has demonstrated that disruption of microvascular integrity permits extravasation of ferumoxytol into the interstitium, enabling detection of hyperacute intramyocardial hemorrhage using T_1_-weighted imaging immediately following reperfusion in a swine model of acute myocardial infarction (Fig. 5) [29].

In addition to blood-pool imaging applications, ferumoxytol enables assessment of myocardial inflammation through delayed macrophage uptake. In patients with acute myocardial infarction, ferumoxytol-enhanced T_2_* mapping has been shown to detect inflammatory activity within infarcted myocardium for up to two weeks after injury, providing complementary information distinct from conventional T_2_-based assessment of myocardial edema and highlighting another potential role for FE-CMR in tissue characterization [59].

### Structural Heart Disease

Structural heart diseases affect both younger patients with CHD and older adults with acquired valvular and degenerative conditions. Ferumoxytol provides high signal-to-noise ratio (SNR) and homogeneous cardiovascular signal during steady-state intravascular distribution, enabling a prolonged acquisition window that supports wide-coverage CMR protocols, simplified acquisition, and comprehensive anatomic and functional evaluation. In this context, FE-CMR has shown increasing translational and clinical utility across a range of structural pathologies, including CHD and valvular heart disease.

Over the past decade, multidimensional FE-CMR methods have been applied to CHD imaging by enabling comprehensive, dynamic assessment of structure, function, and hemodynamics within a single examination. In 2015, the 4D multiphase steady-state imaging with contrast (MUSIC) technique in anesthetized children, generating sub-millimeter, isotropic spatial resolution over multiple cardiac phases [60]. Acquisition time for MUSIC was about 10 min with respiratory gating, and image reconstruction was inline and immediate. Reconstructed images could be viewed in volume-rendered 4D mode, and 2D cines could be reconstructed in arbitrary planes. The derivative ROCK-MUSIC sequence introduced self-gating using rotating Cartesian K-space (ROCK) with compressed sensing, reducing scan time by approximately 40% compared with the prototype MUSIC [61].

**Fig. 6:**
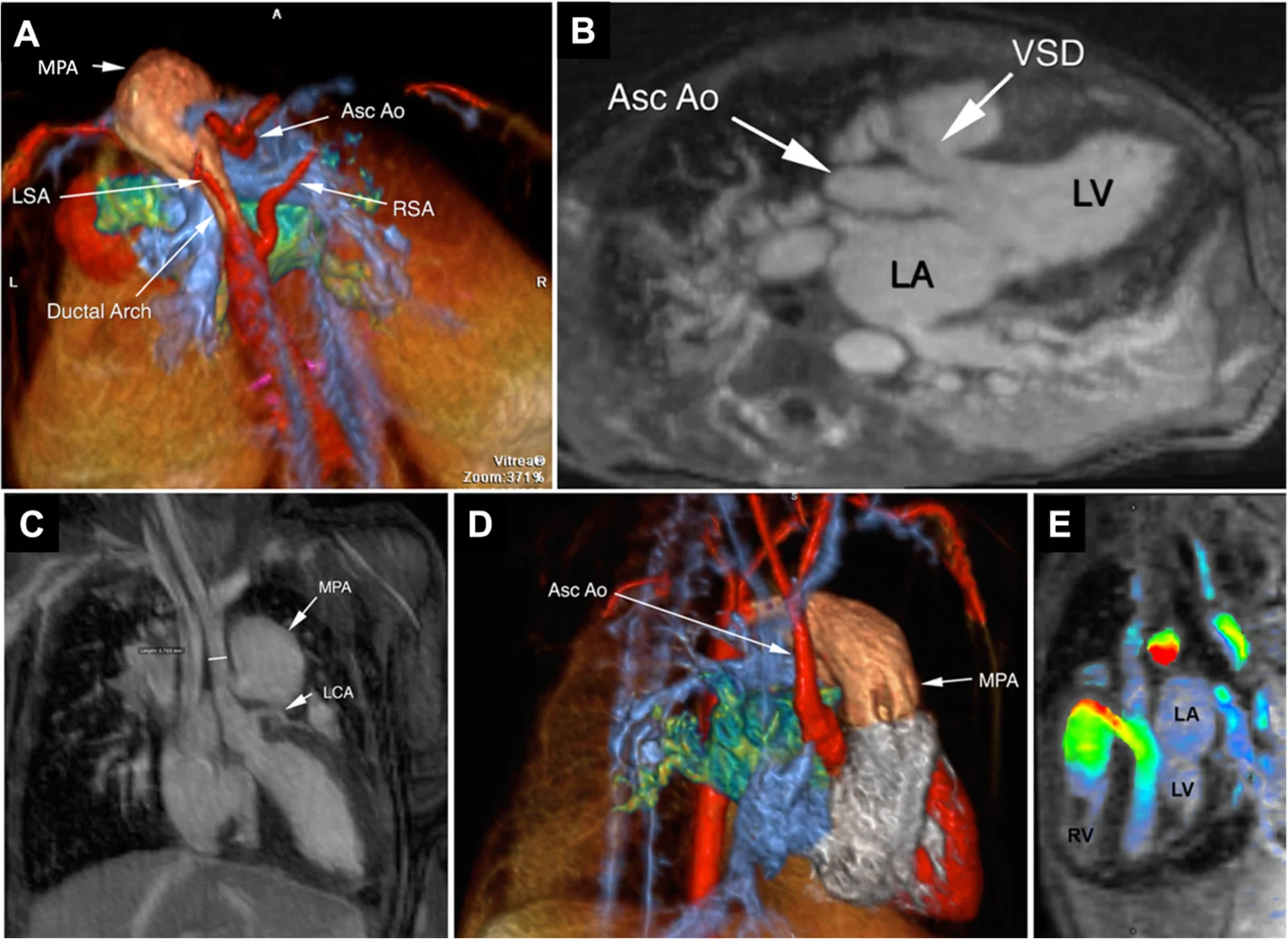
FE-MUSIC Magnetic Resonance Imaging in Interrupted Aortic Arch and VSD. Ferumoxytol-enhanced multiphase steady-state imaging with contrast (FE-MUSIC) was performed to delineate extracardiac vasculature and intracardiac anatomy in a 20-day-old neonate (2.1 kg) with interrupted aortic arch ventricular septal defect (VSD). (**A**, **B**) 3D multiplanar reformatted and color volume-rendered FE-MUSIC images demonstrate the interrupted aortic arch in a bird’s-eye view of the ascending aorta (**A**) and the VSD (**B**). (**C**, **D**) FE-MUSIC images illustrate the spatial relationships among the enlarged main pulmonary artery (MPA), hypoplastic ascending aorta (Asc Ao; 3.8 mm), ductal arch, and intracardiac structures. The proximal course of the left coronary artery (LCA) is well visualized (**C**). Intracardiac anatomy is clearly visualized (**C**). (**E**) 4D-flow imaging demonstrates predominant flow across the VSD. *LA*, left atrium; *LSA*, left subclavian artery; *LV*, left ventricle; *RSA*, right subclavian artery. Reprinted with permission from [62]

FE-MUSIC CMR has had transformative clinical impact, outperforming single-phase, breath-hold ferumoxytol-enhanced cardiovascular MR angiography (MRA) in neonates and infants with CHD referred for clinically indicated imaging [62] (Fig. 6). Initial single-center findings were subsequently validated in a larger, multicenter study, in which FE-MUSIC CMR demonstrated 93% good-to-excellent image quality and 94% diagnostic confidence across 780 intracardiac and vascular connections, while reducing acquisition time from 44 to 12 min compared with local standard MRI in 460 patients with CHD undergoing clinically indicated imaging [36], with volumetry and functional assessment showing strong agreement with conventional multi-slice balanced steady-state free precession (bSSFP) cine imaging [63].

**Fig. 7:**
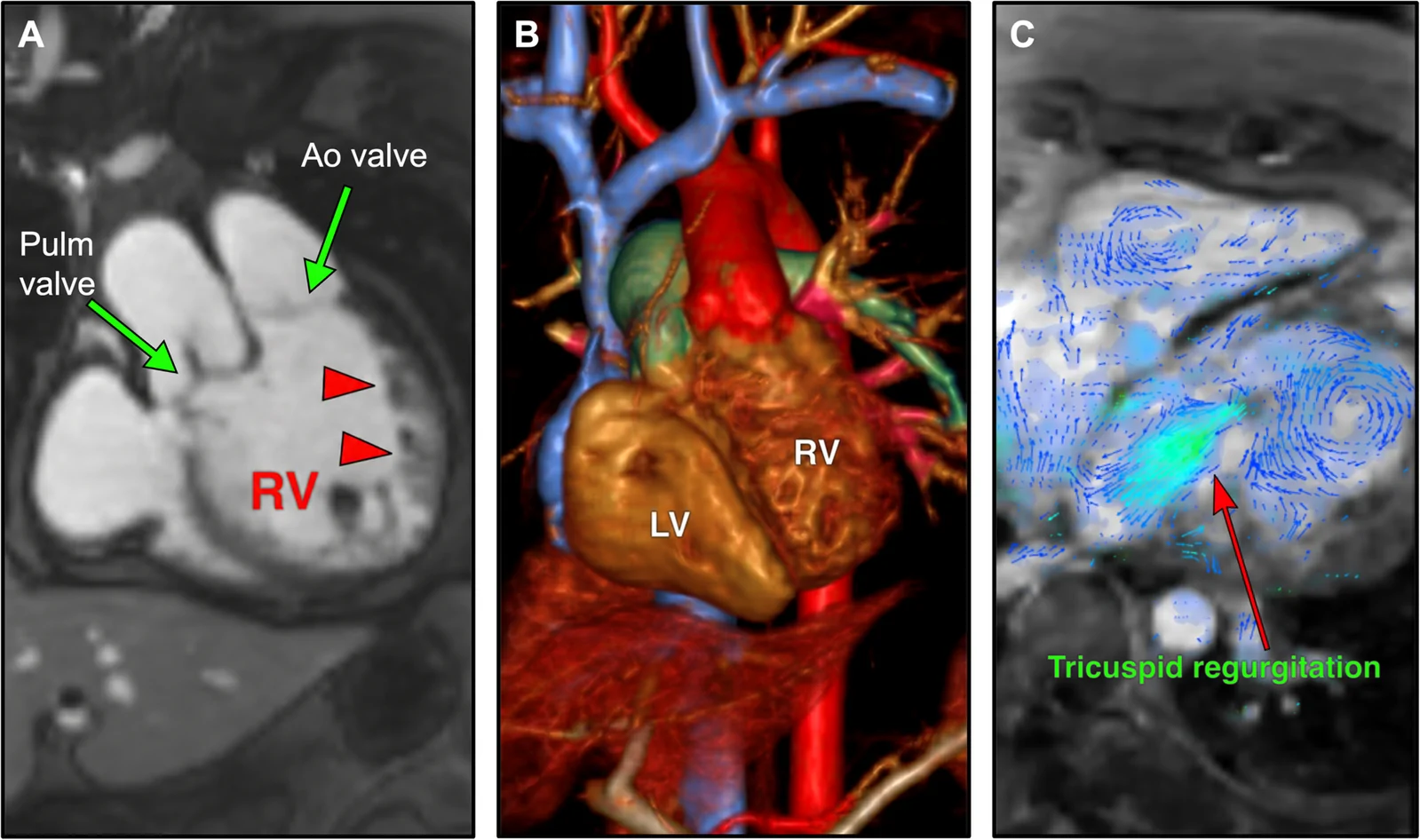
Ferumoxytol-enhanced magnetic resonance imaging in adult congenital heart disease. A 33-year-old female with congenitally corrected transposition of the great arteries and Ebstein anomaly. Following ferumoxytol infusion, 4D MUSIC images were acquired in a single breath-hold, and subsequent 4D-flow images were acquired over 5 min with respiratory gating. (**A**) Frame from a 2D (cine) reformat of MUSIC, showing the trabeculated right ventricle (red arrowheads) and the aortic and pulmonary valves (green arrows). (**B**) Frame from color-rendered 4D MUSIC showing the abnormal position of the trabeculated right ventricle and related anatomy. Systemic arteries and pulmonary veins are rendered in red, pulmonary arteries in green, and systemic veins in blue. (**C**) Frame from 4D-flow reconstructed on the Tempus Cloud, showing a large regurgitant jet (red arrow) from the systemic right ventricle across the tricuspid valve to the left atrium. *Ao*, aortic; *LV*, left ventricle; *MUSIC*, multiphase steady-state imaging with contrast; *Pulm*, pulmonary. RV, right ventricle

Despite these advances, ROCK-MUSIC remains limited by complex motion management and reliance on lengthy offline reconstruction. To address these limitations, recent developments in multidimensional CMR have focused on more efficient motion-resolved acquisition and reconstruction frameworks. Breath-hold FE-MUSIC acquisitions in adult CHD patients have been successfully reconstructed inline within approximately two minutes, with breath-hold durations of 17–24 s and 10–25 cardiac phases, representing a meaningful step toward improved clinical feasibility [64] (Fig. 7). Recently, Zhao et al. extended the ROCK-MUSIC framework to ferumoxytol-enhanced five-dimensional (5D) ROCK-MUSIC by jointly resolving cardiac and respiratory motion using a low-rank tensor reconstruction. This approach represents a step closer to whole-heart imaging in spontaneously breathing neonates and more complex or less cooperative populations [34].

Enabled by over two decades of methodological advances, including self-gating, 3D radial sampling with golden-angle trajectory, and compressed sensing, the 5D frameworks emerged to simultaneously resolve all five dimensions from a single, continuous, ungated acquisition. The prolonged intravascular residence of ferumoxytol provides stable and homogeneous intracardiac signal intensity throughout the entire acquisition, which is critical for robust self-gating and enables reliable extraction of volumetric motion curves across the entire acquisition. Leveraging these properties, SIMilarity-Based Angiography (SIMBA) was introduced. SIMBA clusters continuously acquired k-space data from ferumoxytol-enhanced free-running MRI to identify motion-consistent subsets that can be reconstructed into motion-suppressed 5D whole-heart MRA using a streamlined workflow [65]. Their subsequent compressed-sensing extension extra-dimensional motion-compensation-SIMBA (XD-MC-SIMBA), in combination with ferumoxytol, reduced acquisition times to three minutes or less while preserving image quality in patients with CHD [33]. 5D whole-heart MRA was applied clinically in pediatric CHD patients with ferumoxytol enhancement [66] and achieved diagnostic-quality “one-click” whole-heart imaging in under eight minutes without breath-holding or complex scan planning. However, 5D image reconstruction currently remains offline and time-intensive, and despite growing interest in their clinical applications, these prototype pulse sequences are not yet commercially available and require further validation in larger clinical cohorts.

In addition to advances in multidimensional CMR, several studies have focused on improving reconstruction efficiency and optimizing acquisition strategies in FE-CMR. One study proposed a variable-splitting spatiotemporal network (VSNet) to reconstruct highly undersampled (14-fold acceleration) ferumoxytol-enhanced spoiled gradient echo cardiac cine images [67]. In a cohort of CHD patients, VSNet improved image quality, with significantly higher peak SNR and structural similarity index, more accurate cardiac functional measurements, and superior overall image quality scores. Beyond cardiac function, a recent study highlighted that ferumoxytol-enhanced ultrashort echo time (UTE) CMR outperformed standard bSSFP imaging in stent visualization, thereby informing reintervention planning in CHD patients with intravascular stents [68]. Moreover, ferumoxytol-enhanced 3D UTE enabled whole-heart and vascular angiographic imaging in pediatric patients with CHD in less than one minute, with image quality superior or comparable to conventional 3D bSSFP imaging [69].

**Fig. 8:**
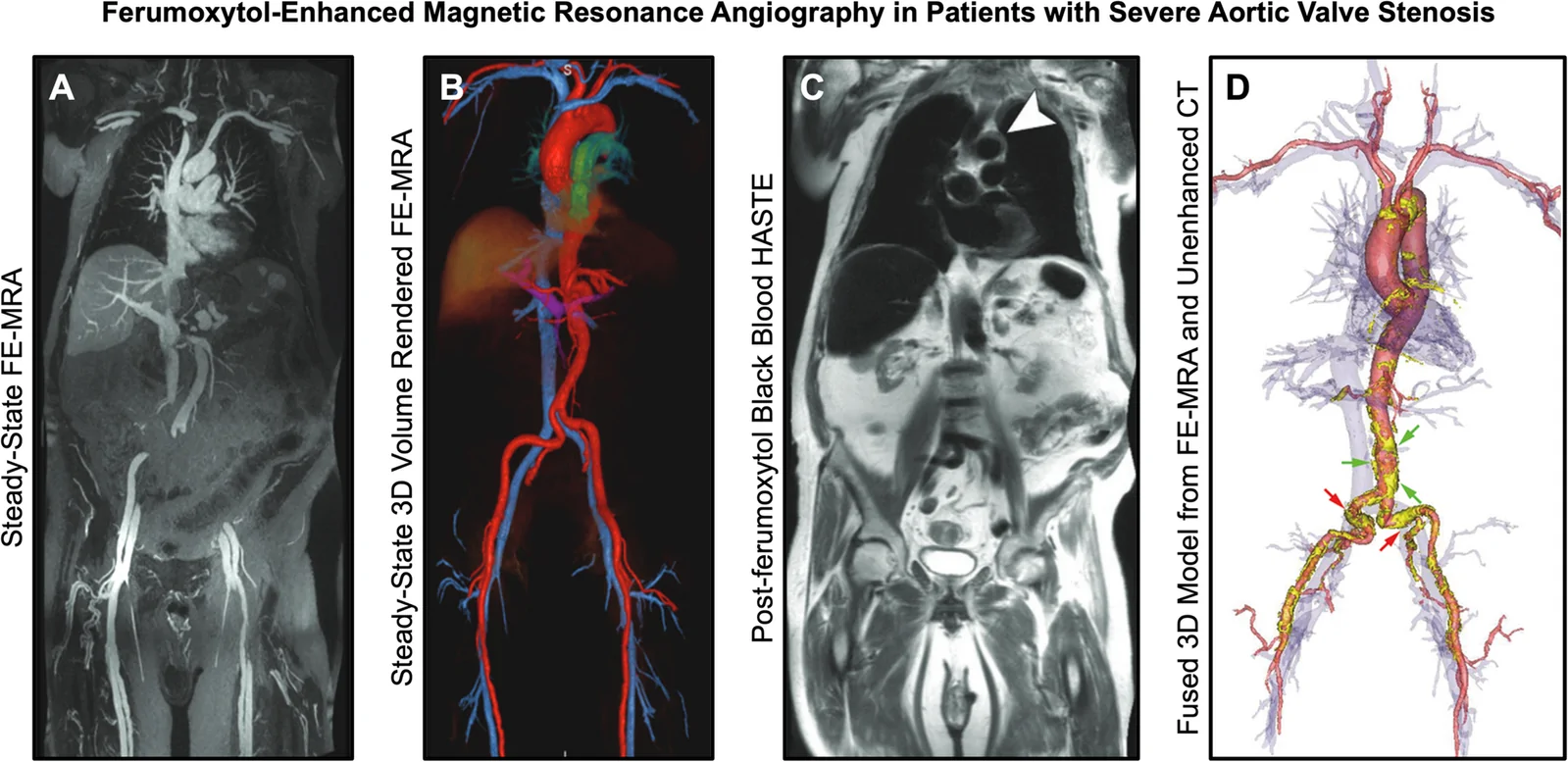
Ferumoxytol-enhanced magnetic resonance angiography for vascular assessment. (**A**, **B**) Coronal steady-state and 3D volume-rendered ferumoxytol-enhanced magnetic resonance angiography (FE-MRA) images acquired at 3.0 T in an 84-year-old male with severe aortic valve stenosis and moderate renal impairment (serum creatinine, 1.7 mg/dL; chronic kidney disease stage 3b), who presented for pre–transcatheter aortic valve replacement evaluation. (**C**) Contrast-enhanced black-blood half-Fourier rapid acquisition with relaxation enhancement image demonstrate atheromatous plaque in the transverse aortic arch (white arrowhead). This patient subsequently underwent successful transfemoral implantation of a 26-mm Edwards SAPIEN valve. Panels **A**–**C** adopted with permission from [70]. (**D**) Fused 3D model reconstruction derived from FE-MRA and unenhanced computed tomography (CT) in an 80-year-old male with severe aortic valve stenosis and moderate renal impairment demonstrates the entire aorta and pelvic access vessels, with atherosclerotic calcifications most pronounced in the iliac arteries. Green arrows indicate calcifications within the abdominal aorta, while red arrows indicate calcifications within the iliac arteries. Panel D adopted with permission from [71]

Valve assessment is also central to structural heart disease evaluation, encompassing both congenital lesions and age-related degeneration. For vascular access planning, FE-MRA has a practical role in pre-transcatheter aortic valve replacement (TAVR), particularly for patients with renal impairment. Researchers have shown that FE-MRA has strong agreement with echocardiography for ventricular outflow tract annular measurement [70], and when fused with computed tomography, can improve vascular visualization and calcified burden assessment [71], and facilitate reliable procedural planning in CKD cohorts [72] (Fig. 8). In addition, ferumoxytol-enhanced 4D-flow MRI has also been shown to support integrated anatomical and hemodynamic assessment of valvular and structural abnormalities, aiding surgical planning and shunt quantification in complex CHD [73].

### Vascular Disease

Contrast-enhanced computed tomographic angiography (CTA) is widely regarded as the reference standard for thoracic vascular imaging, while conventional gadolinium-based MRA is also widely used in clinical practice. Both techniques rely on accurate first-pass bolus timing, and poor timing is the most common cause of poor image quality and diagnostic yield. With ferumoxytol, enhancement of both the arterial and venous systems can be performed at steady-state contrast enhancement. Recent work showed equivalent or superior visualization of the thoracic vasculature in adult patients referred for clinical thoracic FE-MRA compared with dynamic gadolinium-enhanced MRA [31]. Importantly, this approach avoids ionizing radiation and eliminates the need for bolus timing and repeat contrast injections. Emerging data also support its use as a radiation- and gadolinium-free alternative for evaluating pulmonary embolism during pregnancy, when avoidance of GBCA administration may be preferred [35]. In addition to its bright-blood CMR applications, the T_2_* shortening effects of ferumoxytol can be exploited to provide artifact-free black-blood images, facilitating visualization of thrombus and vessel wall [74].

For evaluation of calf arteries, steady-state imaging alone is insufficient. Unlike in the chest, abdomen, and pelvis, where arteries can be readily distinguished from veins based on their distinct anatomy, differentiating diseased calf vessels may be challenging or impossible. Therefore, either non-contrast MRA or first pass MRA of the calf vessels should be performed before steady-state ferumoxytol-enhanced imaging. In due course, it may prove feasible to perform post-processing to separate calf arteries from veins [75], but at the time of writing no practical clinical tools are available to address this purpose.

## Contextualizing Ferumoxytol

CMR has traditionally relied on GBCAs, whose rapid distribution into the extracellular space has enabled transformative advances in tissue characterization, including late gadolinium enhancement and extracellular volume imaging. However, cardiovascular disease is a multiscale process involving abnormalities across multiple biologic compartments, including the extracellular interstitium, intravascular microcirculation, and intracellular environment. As highlighted throughout this review, emerging contrast agents with distinct pharmacokinetic and physiologic properties now offer the opportunity to interrogate these compartments more selectively, expanding the scope of contrast-enhanced CMR beyond conventional extracellular imaging.

Following their initial vascular phase, GBCAs primarily function as extracellular contrast agents, distributing into the interstitial space while remaining excluded from intact cell membranes. This property forms the basis for fibrosis and scar imaging, where delayed accumulation within regions of expanded extracellular space enables characterization of myocardial infarction, focal fibrosis, and diffuse interstitial remodeling. In contrast, ferumoxytol functions as an intravascular tracer that supports assessment of MBV, vascular reserve, hemodynamics, and high-resolution angiographic imaging. Moreover, its prolonged intravascular residence enables continuous, multidimensional, and cardiac phase-resolved imaging paradigms that are difficult or impractical with conventional extracellular contrast agents. Within this framework, ferumoxytol is not simply an alternative to GBCAs, but rather a distinct intravascular imaging approach that complements and expands the physiologic and technical capabilities of CMR. This complementary role was illustrated by Seemann et al., who developed a dual-contrast CMR approach combining ferumoxytol and gadolinium to differentiate intravascular from extravascular pulmonary fluid compartments, enabling quantitative assessment of pathological extravascular lung water in patients with decompensated heart failure [76].

At the intracellular level, manganese-based contrast agents represent another emerging frontier in compartment-specific imaging. As a calcium analog with paramagnetic properties, manganese enters viable cardiomyocytes through voltage-gated L-type calcium channels, enabling direct assessment of intracellular calcium handling and cellular viability [77, 78]. Preclinical studies have demonstrated the potential of manganese-enhanced CMR for quantifying myocardial infarction, detecting myocardial viability, and tracking altered contractility and calcium handling in cardiomyopathies, with potential implications for the development of regenerative heart failure therapies [78]. Although manganese-based imaging remains investigational in cardiovascular applications, it introduces the possibility of directly interrogating intracellular physiology in vivo.

Together, these approaches suggest that the future of contrast-enhanced CMR may evolve toward integrated multicompartmental imaging, in which extracellular, intravascular, and intracellular contrast mechanisms provide complementary information regarding tissue composition, vascular physiology, and cellular function.

## Future Direction

With increasing commercial availability of generic ferumoxytol formulations, the cost of ferumoxytol may decline, potentially improving accessibility and supporting broader clinical adoption. Importantly, multiple recent studies have demonstrated comparable imaging performance between brand-name and generic ferumoxytol across a range of doses and magnetic field strengths, supporting their clinical interchangeability [25, 79]. In parallel, accumulating safety data suggest that the administration risks of ferumoxytol are manageable within appropriately monitored clinical settings [15, 23, 24, 26, 80, 81]. Recent FDA approval of Ferabright for brain MRI represents a major regulatory milestone that may facilitate broader on-label diagnostic applications of ferumoxytol in CMR. These developments, together with the technical advances and emerging applications highlighted throughout this review, suggest that FE-CMR is well positioned to play an expanding role in the future of cardiovascular imaging.

**Fig. 9:**
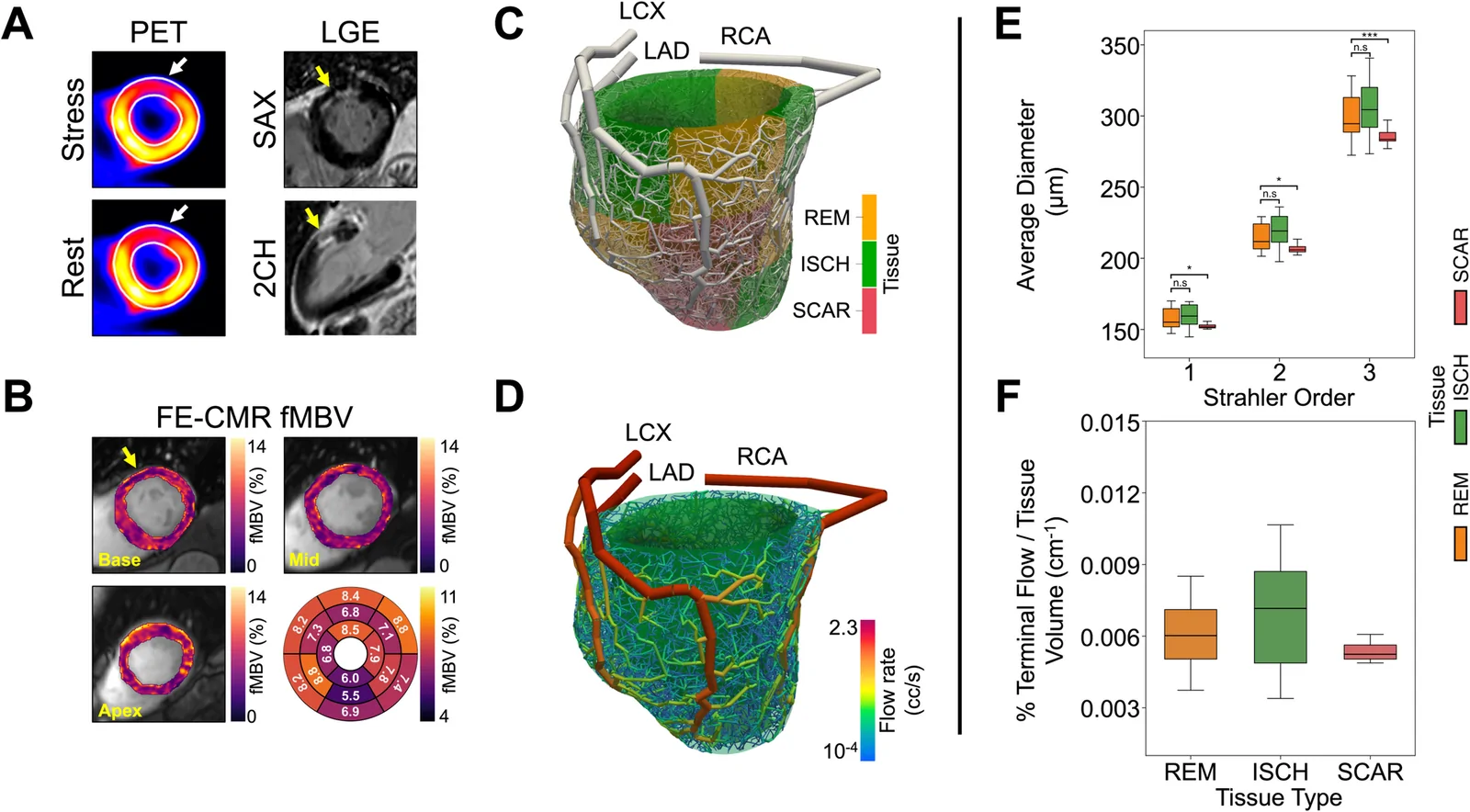
Coronary microvascular network generation using FE-CMR-derived fMBV. (**A**) Representative stress / rest PET myocardial perfusion imaging and LGE images from the same patient demonstrating a fixed perfusion defect in the proximal-to-mid anterior wall (arrows). (**B**) Corresponding proximal, mid-ventricular, and apical FE-CMR-derived fMBV maps with segmental polar plot quantification. The yellow arrows indicate the area of myocardial scar. (**C**) Three-dimensional representation of the constructed microvascular network generated from the same patient shown in (**A**) and (**B**), with labeled myocardial tissue regions. (**D**) Snapshot of flow rate distribution in the arterial tree at the onset of diastole constructed from the same patient. (E) The mean arterial segment diameter for Strahler order numbers 1–3, categorized by each tissue type. (**F**) Variation of the ratio of total coronary flow percentage and tissue volume for each terminal segment in each tissue type (asterisks indicate statistical significance: *p < 0.05, ***p < 0.001; n.s. *p* > 0.05). Adopted with permission from [30]. *2CH*, two-chamber; *FE-CMR*, ferumoxytol-enhanced cardiovascular magnetic resonance imaging; *fMBV*, fractional myocardial blood volume; *ISCH*, ischemic; *LAD*, left anterior descending artery; *LCX*, left circumflex artery; *LGE*, late gadolinium enhancement; *PET*, positron emissions tomography; *RCA*, right coronary artery; *REM*, remote; *SAX*, short axis

Future technical developments will focus on further exploiting the dual-contrast properties of ferumoxytol for a variety of cardiovascular applications. Continued advances in deep learning–based reconstruction frameworks and accelerated acquisition strategies may improve workflow efficiency and facilitate broader clinical implementation of high-dimensional imaging datasets. In parallel, ongoing development of UTE imaging may further expand the utility of FE-CMR in artifact-prone imaging environments [68, 82]. Additional advances at the intersection of FE-CMR and invasive cardiovascular diagnostics and interventions, particularly at lower field strengths, may also offer additional insight. A recent preclinical study demonstrated the feasibility of performing real-time pulmonary artery stent deployment in a swine model using a commercially available low-field 0.55T MRI system with ferumoxytol-enhanced imaging [83]. Real-time gradient echo imaging provided sufficient visualization for catheter navigation, delivery of balloon-expandable stents, and confirmation of post-deployment vessel patency without ionizing radiation. In parallel, preclinical investigations from the same group have demonstrated the feasibility of real-time ferumoxytol-enhanced MRI-guided cardiovascular interventions, including right and left heart catheterization, angioplasty, and stenting, using commercially available low-field MRI systems [84]. In addition to interventional applications, we recently explored the feasibility of constructing subject-specific synthetic coronary arterial networks and noninvasive computation of coronary hemodynamics using an image-based pipeline that integrates ferumoxytol-enhanced coronary MRA with FE-CMR-derived fMBV [30] (Fig. 9). Such approaches may ultimately enable noninvasive derivation of physiologic indices currently obtained primarily through invasive catheterization, including fractional flow reserve and the index of microcirculatory resistance.

## Conclusion

As highlighted throughout this review, ferumoxytol’s unique magnetic and pharmacokinetic properties support quantitative, continuous, multidimensional, and accelerated imaging approaches while expanding access to patient populations in whom conventional CMR may be limited. Clinically, FE-CMR may provide an additional imaging option for patient populations in whom GBCA administration is less desirable based on individualized clinical risk-benefit assessment, particularly in those with impaired renal function, as well as patients who have difficulty tolerating conventional imaging protocols. Collectively, these advances suggest that ferumoxytol is complementary to GBCAs and has strong potential to further expand CMR applications. Continued technical refinement, multicenter validation, and workflow standardization will be essential for broader clinical impact.

## Key References

- Pogosyan A, Keushkerian MJ, Ming Z, Mahmoudi M, Sim MS, Li Y, Finn JP, Nguyen KL. Safety and T_1_ Reactivity for Vasodilator Stress Ferumoxytol-Enhanced Cardiac Magnetic Resonance imaging. *J Cardiovasc Magn Reson*. 2026; 28(1):102,719.
  - ○ This study demonstrates the safety and feasibility of ferumoxytol-enhanced cardiac magnetic resonance imaging with regadenoson stress testing, revealing progressively blunted ferumoxytol-enhanced T_1_ reactivity in remote, ischemic, and infarcted myocardium compared with healthy myocardium.
- Bongiolatti GMR, Prsa M, Romanin L, Si-Mohamed S, Yerly J, Pierzchala K, Tenisch E, Rutz T, Stuber M, Roy CW. Dynamic Whole-Heart MRI of Congenital Heart Disease Patients in the Presence of Turbulent Flow. *J Cardiovasc Magn Reson*. 2026;102,702.
  - ○ Ferumoxytol-enhanced free-running 3D radial ultra-short echo time (UTE) imaging enabled dynamic whole-heart MRI in patients with congenital heart disease, significantly reducing turbulence-related flow artifacts compared with gradient-recalled echo imaging while maintaining high image quality.
- Pogosyan A, Colbert CM, Mahmoudi M, Keushkerian MJ, Llanes AC, Sim MS, Fishbein GA, Chunni Z, Finn JP, Currier JW, Nguyen KL. Ferumoxytol-Enhanced Cardiac Magnetic Resonance for Delineation of Hyperacute Intramyocardial Hemorrhage With Ex Vivo Validation. *Investigative Radiology*. 2026: https://doi.org/10.1097/RLI.0000000000001281.
  - ○ In a swine model of acute myocardial infarction, T_1_-weighted ferumoxytol-enhanced MRI enabled immediate detection of intramyocardial hemorrhage after reperfusion, distinguishing hemorrhagic from non-hemorrhagic infarcts with ex vivo validation.
- Romanin L, Prsa M, Roy CW, Sieber X, Yerly J, Milani B, Rutz T, Si-Mohamed S, Tenisch E, Piccini D, Stuber M. Exploring the Limits of Scan Time Reduction for Ferumoxytol-Enhanced Whole-Heart Angiography in Congenital Heart Disease Patients. *J Cardiovasc Magn Reson*. 2025; 27(1)101,854.
  - ○ This study demonstrates the feasibility of reducing ferumoxytol-enhanced free-running 3D whole-heart angiography acquisition times to 3 min or less while maintaining diagnostic image quality in patients with congenital heart disease.
- Nguyen KL, Ghosh RM, Griffin LM, Yoshida T, Bedayat A, Rigsby CK, Fogel MA, Whitehead KK, Hu P, Finn JP. Four-dimensional Multiphase Steady-State MRI with Ferumoxytol Enhancement: Early Multicenter Feasibility in Pediatric Congenital Heart Disease. *Radiology*. 2021;300(1).
  - ○ This prospective multicenter study demonstrated the feasibility of ferumoxytol-enhanced 4D multiphase steady-state imaging with contrast enhancement (MUSIC) MRI in pediatric congenital heart disease, providing high image quality and diagnostic confidence while reducing acquisition time from 44 ± 20 min to 12 ± 3 min.

## Data Availability

No datasets were generated or analysed during the current study.
